# Actin-Related Protein 4 Interacts with PIE1 and Regulates Gene Expression in *Arabidopsis*

**DOI:** 10.3390/genes12040520

**Published:** 2021-04-02

**Authors:** Wen-Feng Nie, Jinyu Wang

**Affiliations:** Department of Horticulture, College of Horticulture and Plant Protection, Yangzhou University, Yangzhou 225009, China

**Keywords:** *AtARP4*, epigenetic, gene expression, chromatin remodeling

## Abstract

As essential structural components of ATP-dependent chromatin-remodeling complex, the nucleolus-localized actin-related proteins (ARPs) play critical roles in many biological processes. Among them, ARP4 is identified as an integral subunit of chromatin remodeling complex SWR1, which is conserved in yeast, humans and plants. It was shown that RNAi mediated knock-down of *Arabidopsis* *thaliana ARP4* (*AtARP4*) could affect plant development, specifically, leading to early flowering. However, so far, little is known about how ARP4 functions in the SWR1 complex in plant. Here, we identified a loss-of-function mutant of *AtARP4* with a single nucleotide change from glycine to arginine, which had significantly smaller leaf size. The results from the split luciferase complementation imaging (LCI) and yeast two hybrid (Y2H) assays confirmed its physical interaction with the scaffold and catalytic subunit of SWR1 complex, photoperiod-independent early flowering 1 (PIE1). Furthermore, mutation of *AtARP4* caused altered transcription response of hundreds of genes, in which the number of up-regulated differentially expressed genes (DEGs) was much larger than those down-regulated. Although most DEGs in *atarp4* are related to plant defense and response to hormones such as salicylic acid, overall, it has less overlapping with other *swr1* mutants and the *hta9 hta11* double-mutant. In conclusion, our results reveal that *AtARP4* is important for plant growth and such an effect is likely attributed to its repression on gene expression, typically at defense-related loci, thus providing some evidence for the coordination of plant growth and defense, while the regulatory patterns and mechanisms are distinctive from other SWR1 complex components.

## 1. Introduction

Genome-wide transcriptional expression patterns are dynamically regulated in response to various endogenous signals and exogenous stimuli such as cell division and differentiation upon development as well as extreme environmental stresses [1,2]. When plants recognize and receive signals, the signal transduction will cause subsequent changes in transcriptional regulation of relevant functional genes. Transcriptional activation or inhibition is a complicated process that is regulated by many factors. Among them, chromatin modifiers have been extensively studied in both mammals and plants, mainly including chromatin remodeling complexes and histone modifications (e.g., histone variant H2A.Z, histone methylation and histone acetylation) [3,4,5,6]. As shown in *Arabidopsis thaliana*, histone variant H2A.Z and histone acetylation marks are abundant at transcription start sites (TSSs) [7,8,9], which could affect transcription initiation and efficiency. 

The gene expression levels are tightly modulated by chromatin remodeling through managing the density of a packed DNA sequence. As a result, there is a dynamic networked regulation of multiple biological processes by turning “on” or “off” transcriptional regulators and signaling factors. For instance, in plants, the actin-related protein (ARP) containing complexes such as the SWR1 complex and INO80 complex, are essential for vegetative and reproductive development, cell proliferation, endocycle and the programmed cell death [10,11]. These complexes are required for plant organs organization, flowering time, leaf development and senescence as well as floral organ abscission.

ARPs are a large protein family that exists in many eukaryotes [12] and has a moderate degree of sequence similarity between different members [13]. They are integrated into four major nucleosome remodeling complexes and one histone acetyltransferase (HAT), namely INO80.com/INO80, SWR1-C/SRCAP, SWI/SNF/BAF, RSC/PBAF, and NuA4/ TIP60/TRRAP in yeast and humans, respectively [11]. ARPs are a structural component of ATP-dependent chromatin remodeling complex. It can not only promote nucleosomes to move along DNA and expel them from chromatin, but also can change chromatin state through the deposition of histone variants. In budding yeast, there are 10 ARP isoforms, named Arp1–Arp10, with the Arp1, Arp2, Arp3 and Arp10 being predominant in the cytoplasm, and Arp4 to Arp9 being located in the nucleus [11]. The similar subcellular localization of ARPs is also found in *A. thaliana*, where ARP2 and ARP3 form a protein complex [14,15,16]. These ARPs are involved in many basic processes, such as chromatin remodeling, nuclear envelope, histone variant H2A.Z deposition, transcriptional regulation and post-translational modification of histone proteins [3,17,18,19,20]. Specifically, ARP4 is shared by several ATP-dependent chromatin-remodeling complexes and in both yeast and mammals, its functions in these complexes have been well studied. However, little is known about its roles and regulatory mechanisms in plants.

In this study, a component of the SWR1 complex, ARP4, was identified through forward genetic screening for transgenic silencing mutants in *Arabidopsis*. In vitro and in vivo interaction experiments showed that ARP4 could directly interact with the N-terminal of PIE1, a member of the chromatin remodeling SWR1 complex. We then created functionally deficient mutants that stably expressed the *atarp4* mutant allele, and together with the transcriptomic analyses, give a general overview of AtARP4-dependent biological processes, molecular functions, and cellular components. In addition, a low overlapping in differentially expressed genes was shown between AtARP4 and other SWR1 complex components, indicating a specific gene regulatory network for AtARP4. Overall, this study provides feasible materials for the genetic study of AtARP4, and suggests the possible roles of AtARP4 in plant growth and development, and in response to biological and abiotic stresses.

## 2. Materials and Methods

### 2.1. Plant Material and Growth Conditions

For the identification of single nucleotide mutation in *AtARP4*, genetic mapping and gene cloning were performed as described previously [21]. Surface sterilized seeds of *35S:SUC2* wild type (WT) and atarp4-2 mutant were exposed to cold stratification for 3 d, then grown on soil or 1/2 MS medium with 1% glucose at 22/16 °C (day/night), photoperiod (16 h light /8 h dark), 65% relative humidity and 100 μmol m^−2^ s^−1^ photosynthetic photon flux density (PPFD). Seven-day-old seedlings grown on 1/2 MS medium were imaged. The atarp4-2 mutant was backcrossed with WT twice to clean the background. 

### 2.2. Mutant Plant Complementation

For the complementation of *atarp4-2*, *AtARP4* genomic DNA with 2-kb upstream region (as the native promoter region) was amplified from genomic DNA of Col-0 with primers listed in Table S1, and then was cloned into the pENTR/D-TOPO vector (Invitrogen, K240020). The fragment was confirmed by sequencing. Finally, the genomic DNA was subcloned into the pGWB16 vector (with a 4 × Myc tag at the C terminus) using LR clonase II (Invitrogen, 11791020). The construct was transformed into mutants using Agrobacterium tumefaciens GV3101 by dipping the flowers. Leaves in T0 plants growing in the soil were detected by Western bolt with anti-Myc antibody (Millipore, 05-724, Burlington, MA, USA).

### 2.3. Split Luciferase Complementation Assays

For split luciferase complementation assays, leaves of 4-week-old *N. benthamiana* were transformed with *Agrobacterium tumefaciens* GV3101 expressing different proteins of interest. In specific, the coding sequence of the AtARP4 protein was cloned into pCAMBIA-cLUC vector while the N-terminal (1–491 residues) and C-terminal coding regions (492–2055 residues) of PIE1 were cloned into and pCAMBIA-nLUC vector, respectively, and then were transformed into GV3101. The strains were cultured overnight at 28 °C with the pellet being collected after centrifugation and suspended with the incubation buffer (10 mM MgCl_2_, 100 µM acetosyringone, and 10 mM MES, pH 5.6) until OD_600_ reached 1.2. Lastly, an equal volume of suspended culture expressing the indicated nLuc- and cLuc- containing proteins was mixed, and the mixture was then infiltrated into tobacco leaves, and luciferase activity was analyzed at 48 h post-infiltration with a luminescence imaging system (Princeton Instrument). 

### 2.4. Yeast Two-Hybrid Assay

The full-length CDS sequences of *AtARP4*, and two truncated forms (N-terminal and C-terminal) of *PIE1* were cloned into pGADT7-AD or pGBKT7-BD, respectively. Each group of expression vectors was co-transformed into the *Saccharomyces cerevisiae* strain AH109, which were plated on SD minimal media without leucine (Leu) and tryptophan (Trp) (SD2). Transforms from SD2 plates were further transferred to SD-Leu/Trp/His (Histidine) medium with 1mM 3’AT and grown at 30 °C for 3 d.

### 2.5. Total RNA Isolation 

For total RNA preparation, seedlings of 14-day-old WT and *atarp4-2* plants grown on 1/2 MS medium were pooled, frozen in liquid nitrogen and ground. Three biological replicates were performed per genotype with total RNA isolation from 0.1 g tissue was performed using the RNeasy plant kit (QIAGEN, Hilden, Germany) according to the manufacturer’s instructions. The total RNA was dissolved in RNase-free water with the concentration being measured using NanoPhotometer^®^ spectrophotometer (IMPLEN, Westlake Village, CA, USA). The quality was analyzed on 1.2% (m/v) agarose gels and RNA integrity was assessed using the RNA Nano 6000 Assay Kit of the Bioanalyzer 2100 system (Agilent Technologies, Santa Clara, CA, USA).

### 2.6. RNA-seq Analysis

A total amount of 1 µg RNA per sample was used for cDNA synthesis. Sequencing libraries were generated using NEBNext^®^ UltraTM RNA Library Prep Kit for Illumina (NEB, Ipswich, MA, USA) following the manufacturer’s instructions. 

The clustering of the index-coded samples was performed on a cBot Cluster Generation System using TruSeq PE Cluster Kit v3-cBot-HS (Illumia, San Diego, CA, USA) according to the manufacturer’s instructions. Then, the library preparations were sequenced on an Illumina Novaseq platform and 150 bp paired-end reads were generated.

### 2.7. Data Analysis 

For quality control, raw data (raw reads) in fastq format were firstly processed through in-house perl scripts, and then the low-quality reads and reads containing adapter and ploy-N were removed. Q20, Q30 and GC content in the clean data were calculated and all subsequential analyses were carried out based on the clean data.

For reads mapping to the reference genome, HISAT2 (V2.0.5) (accessed on 4 November 2016) was used to build up the index of reference genome and perform the alignment of paired-end clean reads to the reference genome (*Arabidopsis Tair* 10) [22]. The tool can generate a splice junctions database according to the gene model annotation file, thus outputting better mapping results than other non-SPLice mapping tools.

### 2.8. GO Enrichment Analysis

Gene ontology (GO) enrichment analysis was performed using the online tool (http://geneontology.org/) (accessed on 9 October 2020). GO terms with corrected pdaj less than 0.05 were considered as significantly enriched.

### 2.9. Heatmap Plot

Differentially expressed genes (DEGs) were identified based on a false discovery rate FDR < 0.05 and fold change > 2. R (package pheatmap) [23] was used for generation of heat maps and hierarchical clustering; The location map of up-regulated and down-regulated DEGs on chromosomes was generated by TBtools [24].

## 3. Results

### 3.1. Identification of a Novel Allele of ARP4 in A. thaliana

**Figure 1:**
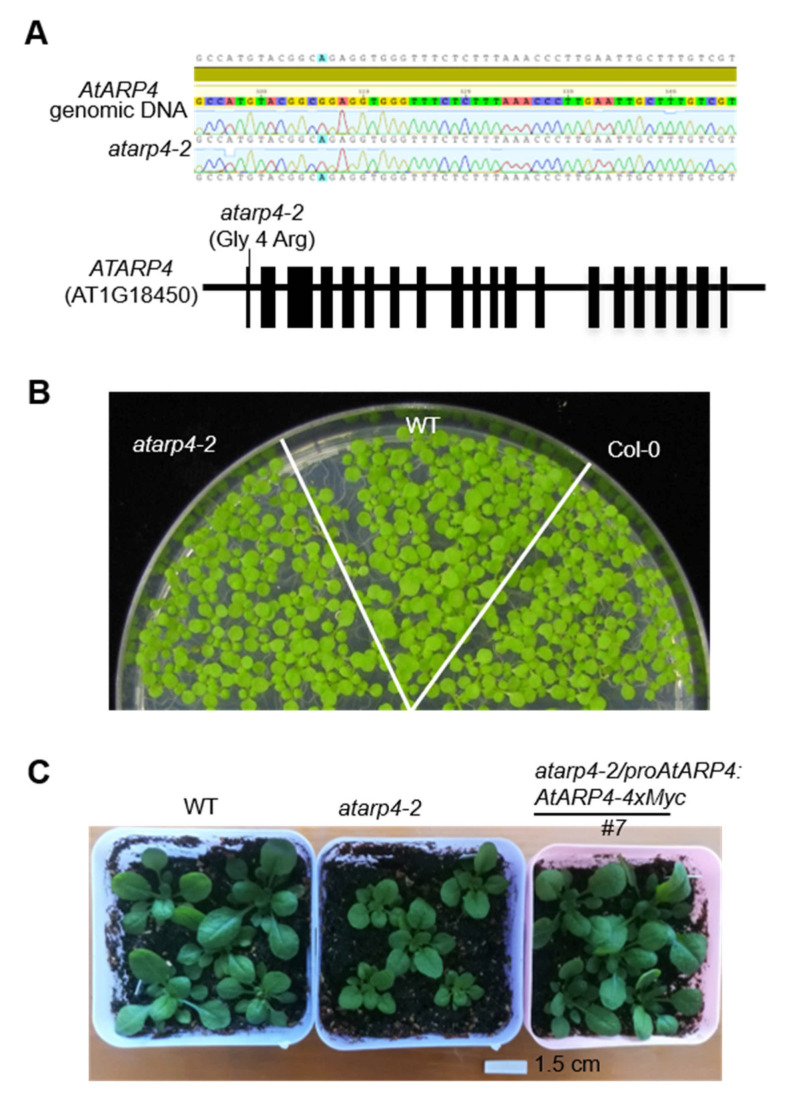
The identification and growth phenotypes of *atarp4-2* mutant allele. (**A**) Sequence chromatograms shows the mononucleotide variation in the *AtARP4* gene (upper panel) and the diagram for AtARP4 displays the precise mutation site and nucleotide substitution (lower panel). The red, black and green boxes indicate the exons, introns, and untranslated regions (UTR), respectively. (**B**) Germination test for *atarp4-2*, wild type (WT), and Col-0. Representative image for 7-day-old seedlings grown on 1/2 MS medium supplemented with 1% glucose. The transgenic plants with expressing cauliflower mosaic virus *35S* promoter-driven sucrose transporter 2 (*35S:SUC2*) were referred as WT. The *atarp4-2* mutant was screened from the *35S:SUC2* background. (**C**) Soil-based growth phenotypes of *atarp4-2* and its complementation line with the *AtARP4* native promoter. Representative images for 5-week-old plants are shown. Western blotting was performed to detect *atarp4-2/proAtARP4:AtARP4-4xMyc* transgenic lines using anti-Myc antibody (Figure S1). Unsegregated T3 plants were identified as homozygous complementation lines and were then used for the growth phenotype detection experiment.

Nuclear actin-related proteins are essential components of chromatin remodeling and modifying complexes that participate in gene expression regulation [10]. Here, based on the previously reported library generated from ethyl methane sulfonate (EMS) mutagenesis [21,25], we performed a forward genetic screen with transgenic *35S:SUC2* plants and identified a mutated allele of *AtARP4* carrying a single nucleotide mutation in the fourth glycine (Gly), which is changed to arginine (Arg) (Figure 1A). To distinguish from the allele with a T-DNA insertion 143 bp upstream of the ATG initiation codon in *AtARP4* (*arp4-1*) reported previously [26], we named this point mutation allele as *atarp4-2*. Germination rate analysis of *atarp4-2* mutant and wild type showed that impairment of *AtARP4* did not affect seeds germination (Figure 1B). However, the subsequent soil-based growth phenotypes displayed substantial differences. In specific, the size of rosette leaves in *atarp4-2* mutant was smaller than that of the wild type (Figure 1C). The expression of *AtARP4-4xMyc* fusion driven by its native promoter restored the phenotype of smaller size of rosette leaves in *atarp4-2* (Figure 1C). This is mostly consistent with the phenotype of *arp4-1* mutant [26], suggesting that ARP4 has a positive effect on plant growth and leaf morphology.

### 3.2. AtARP4 Physically Interacts with PIE1 in Arabidopsis

**Figure 2:**
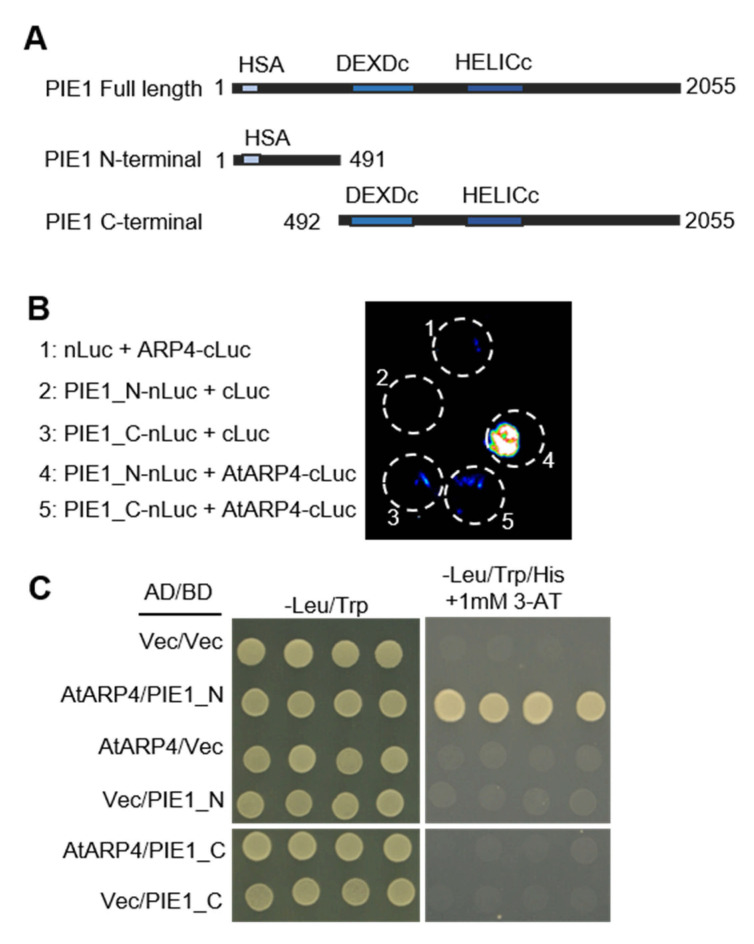
AtARP4 physically interacts with PIE1 in vivo. (**A**) Schematic diagram of truncated forms of PIE1. (**B**) Split luciferase complementation assays for interaction between AtARP4 and PIE1 in vivo. The indicated proteins were transiently expressed as fused to the N-terminal or the C-terminal part of the luciferase protein (nLuc or cLuc, respectively) in *N. benthamiana* leaves. Three independent biological replicates were performed, and one representative result is shown. (**C**) Determination of in vitro interaction between AtARP4 and PIE1 using yeast two-hybrid system. Two truncated forms of PIE1, the N- and C- terminal region were performed. BD, GAL4 binding domain; AD, GAL4 activation domain. “Vec” indicates the empty vector, used as the control.

The association of ARP4 and SWR1 complex has been widely studied in different organisms. In yeast and human systems, ARP4 is shared by several complexes such as INO80 and SWI/SNF (BAF) [18]. Then, using *Arabidopsis* AtARP4 as the protein bait, it was found that all conserved subunits of the SWR1 complex were associated with AtARP4 [27]. In addition, co-immunoprecipitation analysis of plants expressing a *PIE1-3xFLAG-3xHA* fusion protein found that AtARP4 could be detected in the IP products of PIE1 [28]. These results suggest that AtARP4 and PIE1 are associated with each other in *Arabidopsis*. In order to detect whether there is a direct physical interaction between AtARP4 and PIE1, we performed a LCI assay in tobacco leaves. Two truncation forms of PIE1 N-terminal with 1–491 amino acid residues and PIE1 C-terminal with 492-2055 amino acid residues were generated (Figure 2A). We found that AtARP4 could interact with the N-terminal region of PIE1, but not the C-terminal region of PIE1 (Figure 2B). Then, yeast two-hybrid assays were further carried out (Figure 2C). Yeast cells expressing AtARP4 and PIE1-N terminal fragment were able to grow on media deficient in leucine, tryptophan, and histidine, indicating that N-terminal region of PIE1 could directly interact with AtARP4 (Figure 2C). Combined with these results, it confirmed that AtARP4 physically interacted with PIE1 in the SWR1 complex in *Arabidopsis.*

### 3.3. Impairment of AtARP4 Alters Global Transcriptional Regulation

**Figure 3:**
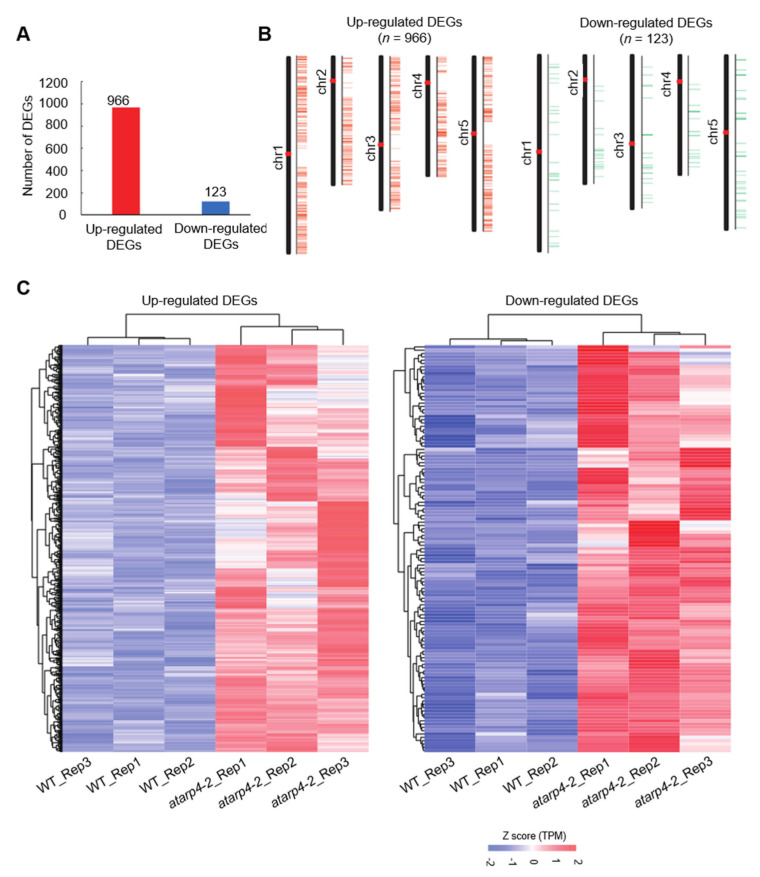
AtARP4 functions in regulating gene expression. (**A**) The number of up-regulated and down-regulated differentially expressed genes in *atarp4-2* mutant. (**B**) The distribution of up-regulated DEGs (left panel) and down-regulated DEGs (right panel) at the five chromosomes of *Arabidopsis* genome. The schematic diagrams were generated by TBtools (Chen et al., 2020). (**C**) Heatmaps for AtARP4-dependent DEGs in WT and *atarp4-2* plants. The heatmap was generated based on the trans per million (TPM) value.

PIE1 has been identified as the scaffold of the SWR1 complex that can promote the deposition of H2A.Z at the whole genome-wide level [28]. Although the SWR1 chromatin remodeling complex is critical to eukaryotic gene expression regulation [29], subunits of this complex such as PIE1, ARP6 and SWC6 as well as H2A.Z are found to play distinctive roles in this process [30]. To elucidate how genes are regulated by AtARP4, global transcriptomic analyses on mRNA isolated from 14-day-old seedlings of WT and *atarp4-2* plants were carried out. The sequencing quality for all samples was examined, and for both WT and *atarp4-2* mutant, the principal component analysis (PCA) showed reliable consistency among the biological triplicates (Figure S2A). Additionally, for each genotype, the Pearson correlation between every two biological replicates was more than 94%, further indicating these biological replicates were highly consistent (Figure S2B). A closer analysis of DEGs in *atarp4-2* mutant compared with WT under non-limiting conditions revealed that the mutation of AtARP4 resulted in remarkable transcriptional changes, among which 966 genes were up-regulated and 123 genes were down-regulated (Figure 3A and Table S2). The number of induced genes was significantly higher than that of down-regulated DEGs by ~7-fold, indicating that AtARP4 may be involved in control of biological processes through negative regulation of expression of related genes. Furthermore, it is interesting to note that most DEGs were distributed on the chromosomes and were far from the centromeric regions (Figure 3B). In addition, heatmaps for both up-regulated and down-regulated DEGs in *atarp4-2* mutant were generated based on the trans per million (TPM) value. It could be clearly seen that the range of alteration in expression levels of down-regulated DEGs was much larger than that of up-regulated DEGs (Figure 3C). 

### 3.4. Identification of Functional Enrichment in atarp4-2 Mutant

**Figure 4:**
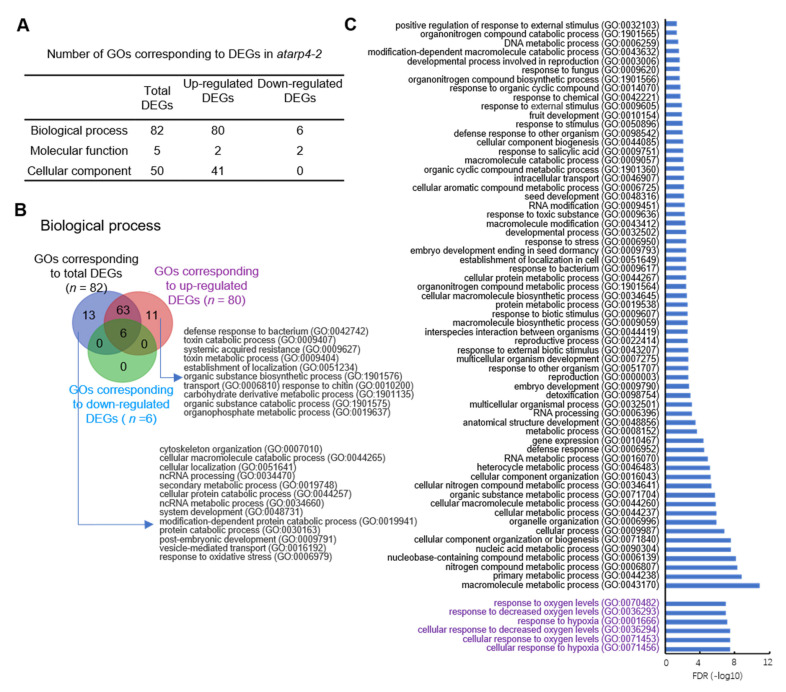
Functional enrichment analysis of DEGs caused by mutation of AtARP4. (**A**) The number of DEGs-based GOs corresponding to biological processes, molecular functions and cellular components in *atarp4-2.* (**B**) The overlap of enriched GOs corresponding to total, up-regulated and down-regulated DEGs in biological process. The detailed GOs were displayed in each subset. (**C**) Significantly enriched GOs shared by total DEGs and up-regulated DEGs in (**B**). The GOs shared by total-DEGs, up-regulated DEGs, and down-regulated DEGs are highlighted by purple.

In order to investigate the functional classification of DEGs identified in *atarp4-2* mutant plants, gene ontology (GO) enrichment analysis was performed. In general, GO enriched pathways were manifested in three aspects including biological processes, molecular functions and cell components, and in total 82, 5 and 50 related pathways were enriched in the total DEGs induced by mutation of AtARP4, respectively (Figure 4A). For the up-regulated DEGs, 80, 2, and 41 pathways were enriched in biological process, molecular functions, and cellular components, respectively, while for the down-regulated DEGs, 6 and 2 pathways were related to biological process and molecular functions, respectively (Figure 4A). 

Then, we closely looked into the enriched pathways identified in DEGs (both up- and down-regulated DEGs), up-regulated DEGs and down-regulated DEGs in *atarp4-2* mutant, and exhibited the specific and overlapped pathways in three aspects as indicated (Figure 4B,C and Figure S3). For the pathways related to biological process, six significantly enriched pathways were common to total DEGs, up-regulated DEGs and down-regulated DEGs, including response to oxygen levels (GO:0070482), response to decreased oxygen levels (GO:0036293), response to hypoxia (GO:0001666), cellular response to decreased oxygen levels (GO:0036294), cellular response to oxygen levels (GO:0071453), and cellular response to hypoxia (GO:0071456) (Figure 4B,C). It was likely to be indicative of the involvement of *AtARP4* in response to changing oxygen levels. Meanwhile, 63 enriched pathways were shared by total DEGs and up-regulated DEGs, which were related to a wide range of biological processes (Figure 4C). For the 11 enriched pathways exclusive to up-regulated DEGs, it was noticeable that most of them were associated with immunity, defense response and metabolic processes (Figure 4B). These included defense response to bacterium (GO:0042742), response to chitin (GO:0010200), systemic acquired resistance (GO:0009627), toxin metabolic process (GO:0009404), organic substance biosynthetic process (GO:1901576), carbohydrate derivative metabolic process (GO:1901135), organic substance catabolic process (GO:1901575), and organophosphate metabolic process (GO:0019637) (Figure 4B). In addition, 13 GOs such as cytoskeleton organization (GO:0007010), ncRNA processing (GO:0034470), system development (GO:0048731), post-embryonic development (GO:0009791), vesicle-mediated transport (GO:0016192), response to oxidative stress (GO:0006979), etc., were only enriched in total DEGs (Figure 4B), suggesting that dysfunction of AtARP4 could both up-regulate and down-regulated these subsets of genes. Similarly, the enriched GOs identified in the indicated clusters of DEGs corresponding to molecular functions and cellular components were also compared (Figure S3). However, there were no pathways overlapped among all three subsets of genes, and only 2 GOs related to RNA binding and 38 GOs associated with organelle and membrane formation were shared by total DEGs and up-regulated DEGs, respectively. In addition, with regard to molecular functions, three specific GOs identified in total DEGs were related to protein binding and nucleoside-triphosphatase activity while two specific GOs in down-regulated DEGs related to nucleic acid binding and oxidoreductase activity were related to protein binding and nucleoside-triphosphatase activity (Figure S3A). For the cellular components, 12 specific GOs in total DEGs were mainly enriched in connective tissue such as cell junction and plasmodesma while only 3 GOs specific to up-regulated DEGs were enriched in vesicle and trans-Golgi network (Figure S3B).

### 3.5. AtARP4 Differs from other SWR1 Complex Components in Gene Regulation

**Figure 5:**
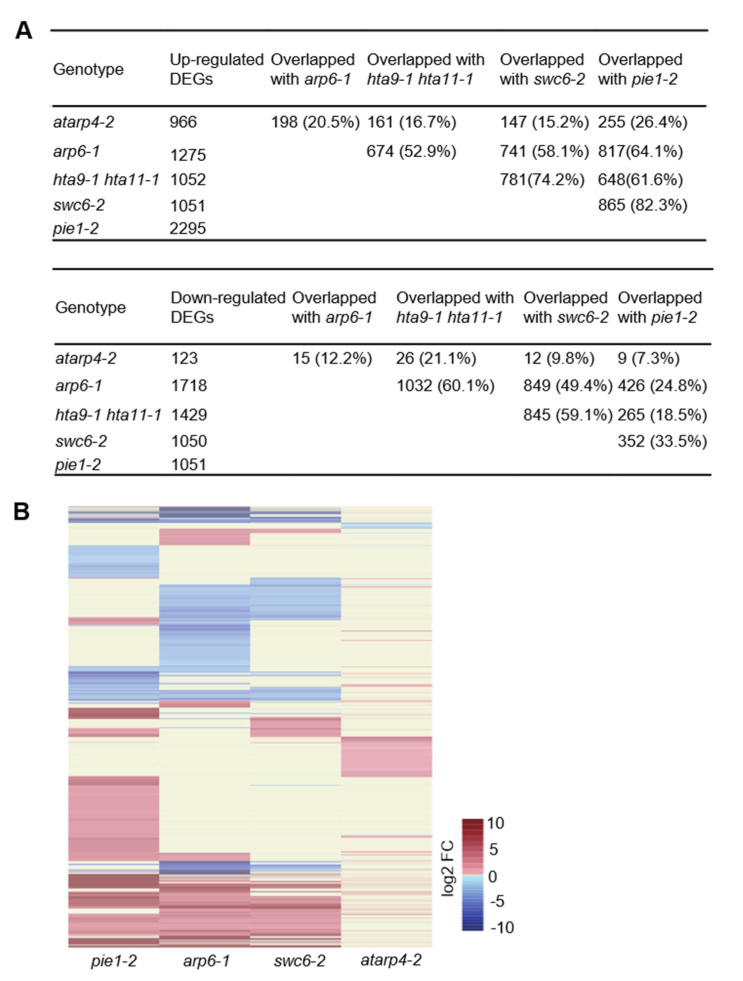
The differential transcriptomic responses between *atarp4-2* plants and *swr1*-related mutants. (**A**) Number of up-regulated and down-regulated DEGs identified in *atarp4-2*, *arp6-1*, *hta9-1 hta11-1*, *swc6-2*, and *pie1-2* mutants, and the respective overlaps of DEGs (fold change ≥ 2 or fold change ≤ 0.5). (**B**) Fold change (FC)-based heatmaps of *pie1-2*, *arp6-1*, *swc6-2*, and *atarp4-2* mutants. The mRNA-seq raw data for *pie1-2*, *arp6-1*, *swc6-2* and *hta9-1 hta11-1* were downloaded from a previous study, Adapted with permission from ref. [3]. 2016 Elsevier.

Unlike other subunits of the SWR1 complex, AtARP4 is shared by multiple remodeling complexes in *A. thaliana,* leading to the complexity of its regulation manner. In order to elucidate the unique roles of AtARP4 and other SWR1 complex subunits such as PIE1, ARP6, and SWC6 in gene regulation, DEGs in respective loss-of-function mutant were compared (Figure 5). As a catalytic subunit of the SWR1 complex, mutation of PIE1 resulted in the largest number of genes reprogrammed, specifically 2295 were up-regulated and 1051 down-regulated [30] (Figure 5A). Compared with other *swr1*-related mutants, *atarp4-2* mutant had the least number of genes differentially expressed, with 966 up-regulated and 123 down-regulated (Figure 5A). Among the 966 up-regulated DEGs, 198, 161, 147 and 255 DEGs were shared with *arp6-1*, *hta9-1 hta11-1*, *swc6-2*, and *pie1-2*, respectively, while, correspondingly, there were 15, 26, 12, and 9 down-regulated genes shared, respectively (Figure 5A). Obviously, there was the least overlaps in the transcriptional responses between mutants of AtARP4 and other SWR1 complex subunits, suggesting that its regulation on gene expression may be in a distinct manner. It could also be clearly seen from the heatmaps for these mutant lines, in which the DEGs were significantly less in *atarp4-2* mutant and the expression pattern was fairly unlike other mutants (Figure 5B). For example, around the middle part of the heatmap where most of up-regulated DEGs in *atarp4-2* mutant converged, the expression of these genes in other mutants of SWR1 complex components had no significant change. Thus, together these results suggested that AtARP4 would have similar roles in controlling the growth and probably the defense response of other subunits of the SWR1 complex in *Arabidopsis* but plausibly through different regulatory networks and mechanisms. 

## 4. Discussion and Conclusions

ARP4 is of great importance to diverse life processes. It could serve as a DNA-length sensor, and play essential roles in maintaining genome stability and regulating gene expression by influencing the structure and environment of chromatin in eukaryotes [17,31,32,33,34,35]. However, it should not be ignored that ARP4 is a common member of several chromatin remodeling complexes, and its multiple identity may greatly broaden its regulation of cellular processes, but also increase the complexity of regulatory mechanisms. Despite the accumulating evidences showing the association of ARP4 with other members of SWR1 complex in different organisms [18], here using the LCI and Y2H assays, we confirmed the direct physical interaction between AtARP4 and the platform protein PIE1 in *Arabidopsis*. Since AtARP4 is closely related to other SWR1 complex proteins, it is not surprising that they play a similar role in some cellular processes. As was shown in this study, the introduction of a novel point mutation in AtARP4 led to growth retardation and altered leaf development and morphology, which were broadly consistent with growth defects caused by T-DNA insertion or RNA interference mediated suppression of AtARP4 expression [26]. More importantly, similar growth defect phenotypes were also found in mutants of SWR1 complex subunits and *h2a.z* mutant [19,28,36,37,38]. These results, to some extent, strengthened the understanding that members of SWR1 complex were involved in the regulation of plant growth and development. As the growth and development of single mutant of AtARP4 and SWR1 complex subunits were not seriously inhibited, there raised the questions are raised about whether AtARP4 and SWR1 complex subunits had partial redundant function and whether their effect on growth was dose-dependent, issues which needed to be further verified.

Plant growth and development are complicated events that can be influenced by environmental factors and genetically or epigenetically regulated. The progress of this basic life process is always accompanied by large-scale transcriptomic reprogramming, which requires correct initiation, elongation, maintenance of transcription and so on [39,40]. Successful gene expression largely depends on proper chromatin organization and structure, which can be modulated and accomplished by chromatin remodeling complexes, such as SWR1, NuA4 and INO80 complexes. Thus, a properly functioning chromatin remodeling complex is essential for plant growth, development, and gene expression. However, as a shared subunit of multiple chromatin remodeling complexes, no completely knock out mutant for *AtARP4* has been identified so far. In fact, a T-DNA insertion in the promoter region of *AtARP4*, specifically 143 bp upstream of the transcription start site (TSS), had resulted in a remarkable drop by 35–40% in AtARP4 protein levels and also partial sterility [26]. These results led us to propose that AtARP4 knockout mutants may be lethal. Here, by EMS mutagenesis and forward genetic screening, the *atarp4-2* mutant allele was isolated and it had caused severe growth retardation and misregulated gene expression. Since the enriched GOs of a large number of differentially expressed genes in *atarp4-2* mutant under controlled conditions were related to diverse biological processes, molecular functions, and cellular components, *atarp4-2* mutant allele could be an excellent resource for the exploration of biological functions of AtARP4 and also the corresponding gene regulation patterns. 

It was shown that around 2000~3000 genes were reprogrammed at transcriptional levels due to respective impairment of PIE1, ARP6 and SWC in *Arabidopsis* [30], which further supported the importance of the SWR1 chromatin remodeling complex in regulating eukaryotic gene expression. Unlike these mutants, *atarp4-2* mutant had a smaller number of DEGs (Figure 5A). Additionally, there was a lower degree of overlapping of up-regulated or down-regulated DEGs between *atarp4-2* mutant and *swr1*-related mutants (Figure 5B). These results indicated AtARP4 plausibly function in a distinct manner. This is also understandable because of the specificity of AtARP4 present in multiple complexes. As an integral subunit of different chromatin remodeling complexes, AtARP4 not only needs to cooperate with other SWR1 complex proteins to regulate the expression of growth-related genes, mainly in a H2A.Z-dependent manner [33], but also is required to balance and assist different chromatin remodeling complexes, which may simultaneously play different regulatory mechanism roles. Therefore, as a linker of different chromatin remodeling complexes, AtARP4 is more likely to play an auxiliary role. Furthermore, there were significantly more genes up-regulated than those down-regulated, suggesting AtARP4 may mainly function as a suppressor of gene expression at the genome-wide level. It was partly evidenced by the fact that Arp4 was able to form a heterodimer with monomeric actin in chromatin remodeling complexes and suppress the nuclear F-actin formation in mammalian cells [18]. In addition, histone acetylation is one of the post-translational modifications contributing to the establishment of correct chromatin environment. It has been known that ARP4 and the histone acetyltransferases (HATs) constitute the NuA4 (nucleosome acetyltransferase of H4) complex, while Alp5, the homolog of BAF53/Arp4 in fission yeast is also required for histone H4 acetylation [41], indicating that ARP4 is essential for the H4 acetylation. Given the important role of histone acetylation in gene activation, ARP4-mediated histone acetylation (such as H4 acetylation) is of interest in the exploration of ARP4-dependent gene regulatory mechanisms. Regardless, it also should be noted that samples of ARP4 and SWR1-related mutants for transcriptomic analysis were not collected under the same conditions, that is the different growth conditions, sampling time, and sequencing methods may lead to low DEGs overlaps, and that the possibility that a subset of DEGs in *atarp4-2* mutant plants induced by other mutations could not be completely excluded, even though this mutant was backcrossed three times with the WT plants.

In addition to the involvement in regulating gene expression, *AtARP4*-dependent DEGs are enriched in GOs related to plant defense and response to stimuli (Figure 4C), suggesting that AtARP4 also plays essential roles in coping with stresses. Additionally, we identified six enriched GOs corresponding to the pathways related to oxygen levels and hypoxic stress (Figure 4C). Hypoxic stress, frequently caused by flooding events, disrupts the oxygen diffusion in plant tissues and thus leads to cellular hypoxia, decreases in ATP production, and a burst in the generation of reactive oxygen species (ROS) [42]. ROS and redox state are essential to the response to biotic stress [43,44,45]. These results suggest that the genes corresponding to oxygen levels and hypoxic stress are differentially induced by AtARP4, and that plants with mutation of ARPs may be more sensitive to biotic stimulus. As stationary organisms, plants often suffer from unfavorable or extreme environmental conditions, therefore, it is of great significance to reveal the regulatory pathways of AtARPs in response to biotic and abiotic stresses. What is more importantly, similar defensive responses have been demonstrated in other SWR1 complex members [30].

## Data Availability

The DEGs presented in this study are available in supplementary materials.

## Supplementary Materials

The following are available online at https://www.mdpi.com/article/10.3390/genes12040520/s1, Figure S1: Western blot detection of AtARP4-4xMyc in the T0 transgenic lines; Figure S2: Quality control the mRNA-seq data; Figure S3: The overlap of enriched GOs corresponding to the total, up-regulated and down-regulated DEGs in molecular function(A) and in cellular component (B); Table S1: List of primers used in this study; Table S2: List of differentially expressed genes up-regulated or down-regulated in *AtARP4* knockout mutant line.
