# Supplementary material for: Actin-Related Protein 4 Interacts with PIE1 and Regulates Gene Expression in Arabidopsis
Source: Genes (Basel). 2021 Apr 2;12(4):520. doi: 10.3390/genes12040520 (PMC8066076; doi:10.3390/genes12040520)
Supplement: Supplementary file 1 [file genes-12-00520-s001.zip › Supplemental Figs 1-3 and Table S1.pdf]

Figure S1

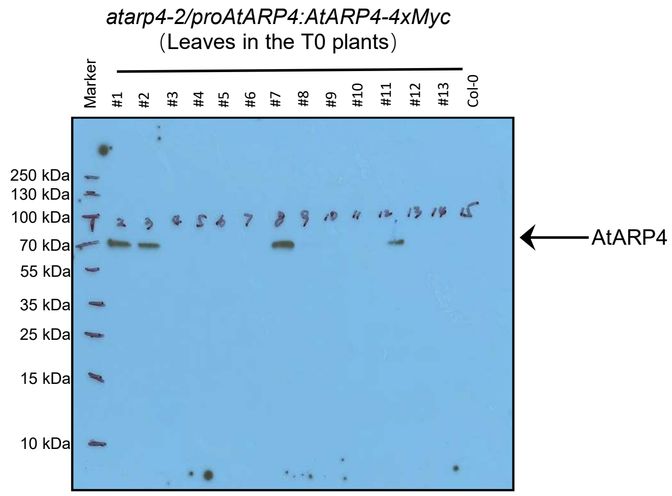

Supplemental Figure 1. Western blot detection of AtARP4-4xMyc in the T0 transgenic lines. The number marked in the image represents the lane number. The protein marker was marked in the image.

Figure S2

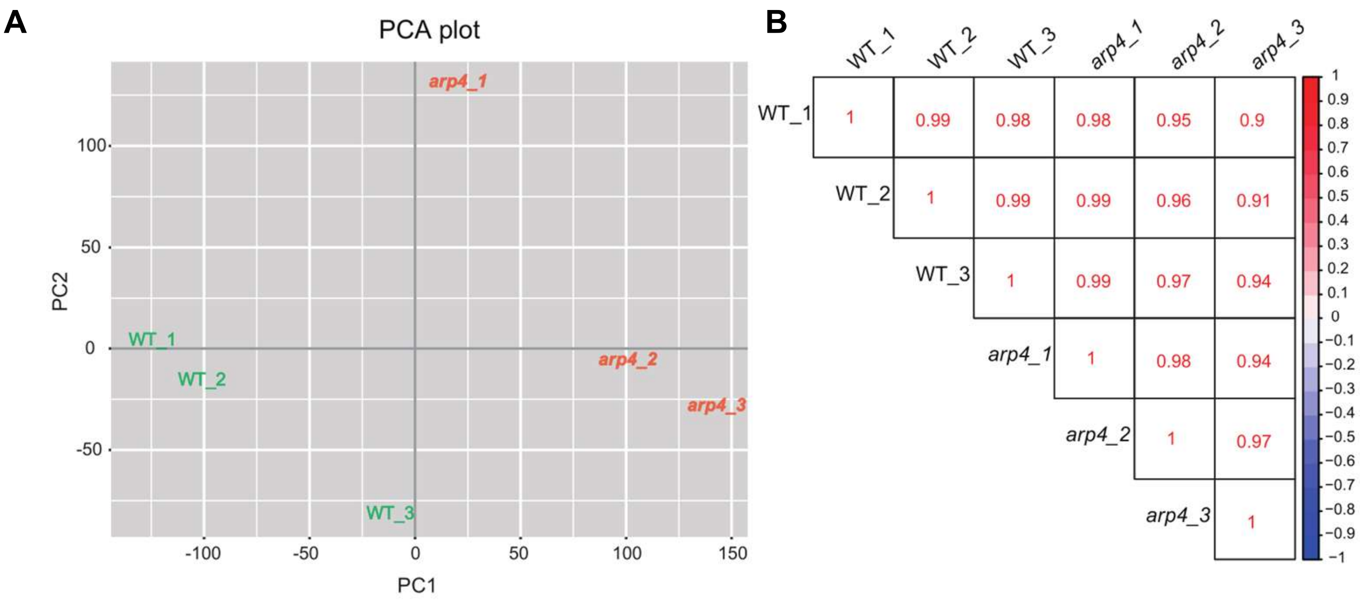

Supplemental Figure 2. Quality control the mRNA-seq data.
The principal component analysis (PCA) (A) and Pearson correlation index (B) indicate the consistency among the three biological replicates for each genotype.

Figure S3

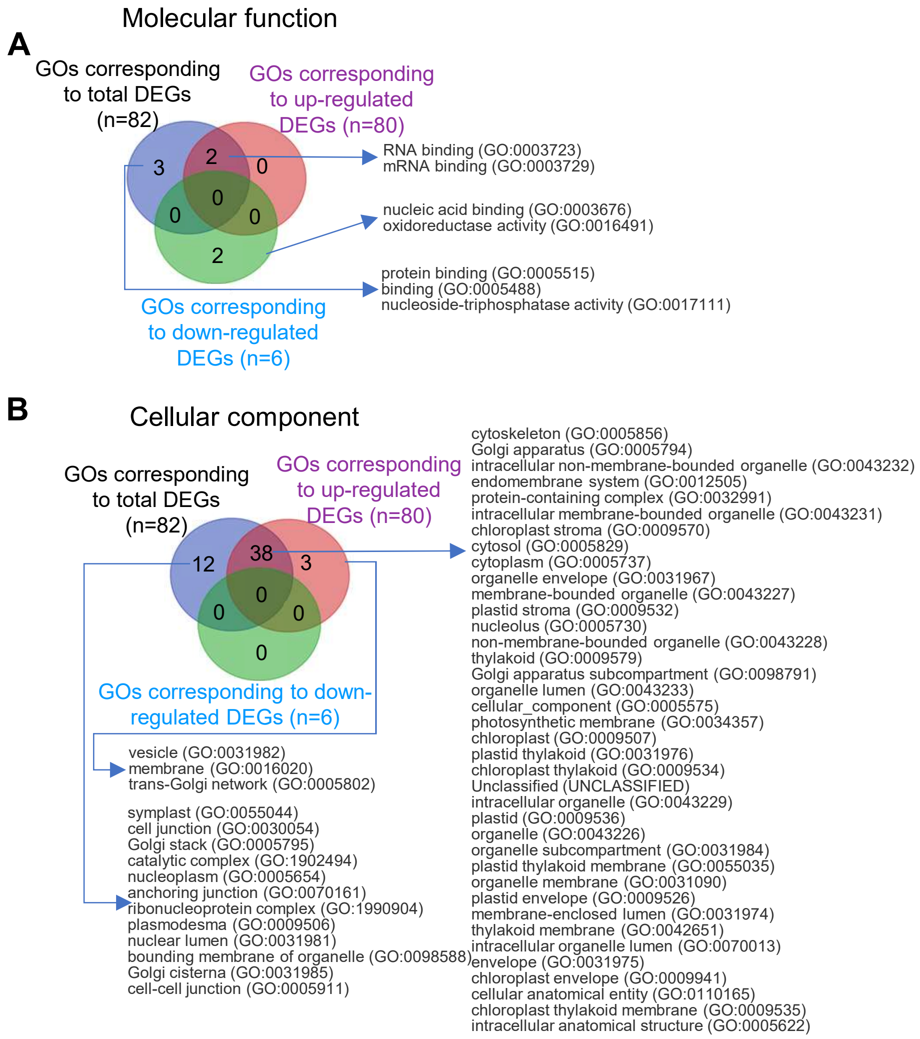

Supplemental Figure 3. The overlap of enriched GOs corresponding to the total, up-regulated and down-regulated DEGs in molecular function(A) and in cellular component (B).

Supplemental Table 1. List of primers used in this study

Primer name	Sequences (5′→3′)	Purpose
pEnter-AtARP4-F	CACCCGAATTCATTCTCCTCTCAA	Topo cloing
pEnter-AtARP4-R	AGGGCATTTTCTCTGAATGT	Topo cloing
AtARP4-seq1	ACCGATTATGCTGGCTAAACC	Sequencing
AtARP4-seq2	TCCGATGAGCTCACTTACAGAC	Sequencing
AtARP4-seq3	GCCAATTTTGTTCTCCGCCA	Sequencing
AtARP4-seq4	GGCGGAGAACAAAATTGGCT	Sequencing
AtARP4-seq5	GGTCCCCACAACTCGAACTC	Sequencing
AtARP4-seq6	CGGAGGGTTTAAACACGGGA	Sequencing
AtARP4-seq7	TGGGGCAGTAGATGGTGTAGA	Sequencing
AtARP4-seq8	TCTACACCATCTACTGCCCCA	Sequencing
AtARP4-seq9	TTTTGCTACTGGGCGTGCTA	Sequencing
AtARP4-seq10	ACAGCATATAGAGCAGAAGTGGA	Sequencing
AtARP4-seq11	TTCCTGACACCACGGAAAGC	Sequencing
AtARP4-seq12	GGGAGTATCAGGAACTCGACA	Sequencing
AtARP4-seq13	AAGGACGCTCGAGATTGGTG	Sequencing
AtARP4-seq14	CGAGGCTCAAAACGCTCAAG	Sequencing
AtARP4-seq15	TTATGCCAGCTTGCTGGTGG	Sequencing
